# Optical manipulation of the alpha subunits of heterotrimeric G proteins using photoswitchable dimerization systems

**DOI:** 10.1038/srep35777

**Published:** 2016-10-21

**Authors:** Gaigai Yu, Hiroyuki Onodera, Yuki Aono, Fuun Kawano, Yoshibumi Ueda, Akihiro Furuya, Hideyuki Suzuki, Moritoshi Sato

**Affiliations:** 1 Graduate School of Arts and Sciences, The University of Tokyo, Komaba, Meguro-ku, Tokyo 153-8902, Japan

## Abstract

Alpha subunits of heterotrimeric G proteins (Gα) are involved in a variety of cellular functions. Here we report an optogenetic strategy to spatially and temporally manipulate Gα in living cells. More specifically, we applied the blue light-induced dimerization system, known as the Magnet system, and an alternative red light-induced dimerization system consisting of *Arabidopsis thaliana* phytochrome B (PhyB) and phytochrome-interacting factor 6 (PIF6) to optically control the activation of two different classes of Gα (Gα_q_ and Gα_s_). By utilizing this strategy, we demonstrate successful regulation of Ca^2+^ and cAMP using light in mammalian cells. The present strategy is generally applicable to different kinds of Gα and could contribute to expanding possibilities of spatiotemporal regulation of Gα in mammalian cells.

Heterotrimeric guanine nucleotide-binding proteins (heterotrimeric G proteins) are made up of alpha (Gα), beta (Gβ) and gamma (Gγ) subunits. They act as molecular switches inside cells, and their main function is signal transduction working together with G protein-coupled receptors (GPCR). Gα dissociates from the Gβγ dimer when responds to a ligand-induced conformation change of GPCR, and then activates effector proteins in particular signal transduction pathways, evoking downstream signaling cascades^1^.

To achieve precise manipulation of Gα, chemically-inducible dimerization (CID) system^2^ has been applied previously. Putyrski and Schultz developed a rapamycin-based system to bypass the GPCR by direct activation of Gα, which induces downstream signaling cascades. They demonstrated that the plasma membrane recruitment of constitutively active form of Gα_q_ (herein after referred to as Gα_q_) and Gα_s_ results in the activation of their effectors^3^,^4^. Despite the achievement gained through the robust protein-protein interaction using CID system, it hardly reaches spatiotemporal control because of the irreversibility and the diffusiveness of the chemical dimerization. On the other hand, optogenetic tools like optoXRs^5^, which allow achieving high spatiotemporal precision, have been developed for manipulation of Gα. OptoXRs are composed of an extracellular component derived from light sensitive rhodopsin and an intracellular component derived from GPCR. A limitation of optoXRs is that rhodopsin gets broad absorption band. Practically, fluorescent tools used as biomarkers or biosensors are commonly required to evaluate the effect of the optogenetic perturbation on the cell. However, optoXRs are often incompatible with these tools because the excitation wavelength of the fluorescent tools may potentially perturb the function of optoXRs having broad absorption band.

In this study, we certify a strategy that Gα achieves activation via light-controlled translocation of Gα from the cytoplasm to the plasma membrane using photoswitchable dimerization systems. In contrast to optoXRs, this strategy affords unique advantage in selectable usage of photoswitchable dimerization systems having narrow absorption spectrums, thereby allowing the feasibility of combinational application with other fluorescent tools. Additionally, this strategy is generally applicable to different classes of Gα. To be more specific, we report individual approaches to manipulate Gα_q_ and Gα_s_ based on the Magnet system and the PhyB/PIF6 system, and we show the light-dependent regulation of two second messengers: Ca^2+^ and cAMP.

## Results

### Design scheme

**Figure 1:**
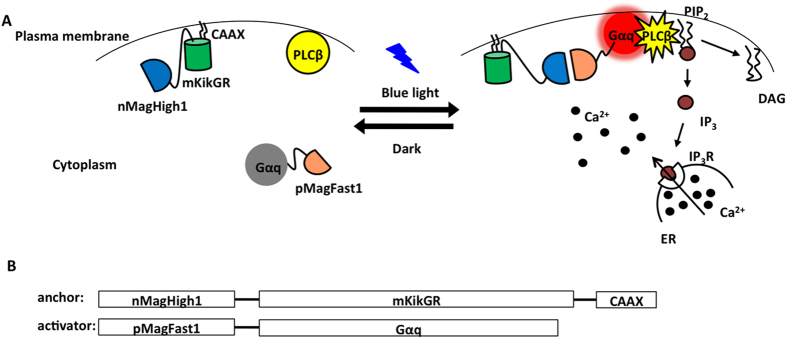
The present approach for manipulating Ca^2+^ via Gα_q_ using the Magnet system. (**A**) Light-inducible dimerization of the Magnet system allows Gα_q_ to translocate to the plasma membrane and trigger cytosolic Ca^2+^ release through the phospholipase C-beta (PLCβ)-inositol triphosphate receptor (IP_3_R) pathway. (**B**) Constructs for nMagHigh1-mKikGR-CAAX and pMagFast1-Gα_q_.

Magnet system consisting of nMagHigh1 and pMagFast1 is a recently developed dimerization system^6^, which is based on a photoreceptor VIVID derived from *Neurospora crassa*^7^,^8^,^9^. Heterodimerization occurs between nMagHigh1 and pMagFast1 upon blue light illumination (Fig. 1A). The Magnet system shows high interaction affinity and fast switch-off kinetic, absorbs a narrow spectrum of blue light (Supplementary Fig. S1) and its heterodimerization can be induced with low light power. These facilitative properties of the Magnet system show the feasibility to gain precise spatiotemporal control of Gα. In utilization of the photoswitchable Magnet system, we designed a strategy that blue light induces Gα_q_ to move from the cytoplasm toward the plasma membrane, where Gα_q_ activates the effector phospholipase C-beta (PLCβ) and triggers the cytosolic Ca^2+^ release (Fig. 1A).

In this translocation-based approach, pMagFast1 linked with Gα_q_ at the C-termini is localized in the cytoplasm, while nMagHigh1 tagged with mKikGR is anchored to the plasma membrane using a membrane localization sequence (CAAX motif)^10^. The fluorescent protein mKikGR can be used to monitor the expression level of the probe and mark the cells of interest. After blue light stimulation, heterodimerization occurs between nMagHigh1 and pMagFast1, and thereby induces the translocation of pMagFast1-linked Gα_q_ from the cytoplasm to the plasma membrane. The translocation of Gα_q_ to the plasma membrane activates the downstream effector PLCβ, which produces inositol 1,4,5-triphosphate (IP_3_) and diacylglycerol (DAG). Subsequently, IP_3_ binds to IP_3_ receptor (IP_3_R) on endoplasmic reticulum (ER) and leads to the release of Ca^2+^ from the intracellular store sites to the cytosol^3^,^11^,^12^.

### Blue light-induced membrane recruitment of Gα_q_ using the Magnet system

Translocation to the plasma membrane induced by the dimerization of nMagHigh1 and pMagFast1 is a prerequisite for the activation of Gα_q_. To definitively ascertain the interaction between nMagHigh1 and pMagFast1 upon blue light stimulation, we performed the cell-based translocation assay using a total internal reflection fluorescence (TIRF) microscope. nMagHigh1-mKikGR-CAAX and DsRedEx2-pMagFast1-Gα_q_ were coexpressed in COS-7 cells. nMagHigh1 was targeted to the plasma membrane while pMagFast1 was targeted to the cytoplasm. We traced the fluorescence intensity of DsRedEx2 in the plasma membrane and observed a remarkable increase of TIRF signal directly after 488 nm laser illumination (Supplementary Fig. S2A). In order to verify the light dependency of the fluorescence change, we introduced C71S^8^ mutation to nMagHigh1 and pMagFast1, which impairs the photoswitching dimerization of these two proteins. As expected, the C71S substitutions generated scarcely TIRF signal change upon 488 nm light illumination (Supplementary Fig. S2B). These results demonstrate that the dimerization of nMagHigh1 and pMagFast1 is switched on upon blue light illumination, and this blue light-dependent dimerization can induce the recruitment of cytosolic DsRedEx2-pMagFast1-Gα_q_ to the nMagHigh1-decorated plasma membrane.

### Blue light-induced Ca^2+^ release

**Figure 2:**
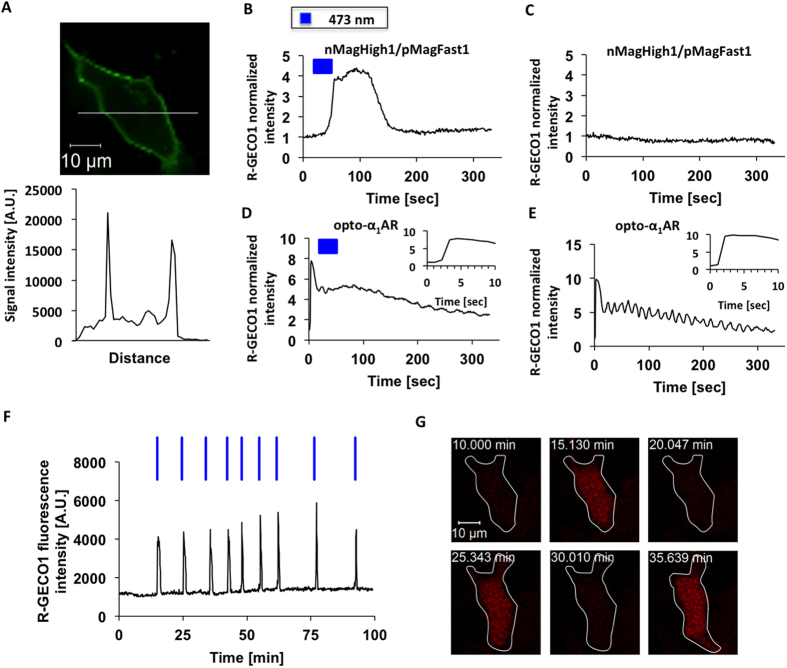
Control of Ca^2+^ release with blue light and comparison with opto-α1AR. (**A**) Subcellular localization of nMagHigh1-mKikGR-CAAX at the plasma membrane (upper panel) and its line profile (lower panel). (**B–E**) A comparison between the present approach based on the Magnet system and conventional opto-α1AR. For Ca^2+^ imaging, the cells were expressed with R-GECO1 and continuously exposed to excitation light at 559 nm. In the Magnet-based approach, the response of R-GECO1 was observed when the cells were stimulated with activation light at 473 nm (**B**) but not observed in the absence of the activation light (**C**); In opto-α1AR, the response of R-GECO1 was observed with (**D**) or without (**E**) blue light illumination at 473 nm, suggesting the excitation light of R-GECO1 at 559 nm activates opto-α1AR. The insets are partially enlarged views in Figure D and E. (**F,G**) Ca^2+^ imaging using the Magnet based approach with pulsed blue light illumination. Time-lapse fluorescence imaging showed that repeatable response of R-GECO1 was evoked upon activation light at 473 nm. Blue bars indicate 473 nm laser illumination.

nMagHigh1-mKikGR-CAAX and pMagFast1-Gα_q_ were transfected to the human embryonic kidney 293 (HEK 293) cells. As confirmed by the fluorescence of mKikGR, nMagHigh1 domain was anchored to the plasma membrane (Fig. 2A). To quantify the Ca^2+^ level induced by activated Gα_q_, we transfected a Ca^2+^ indicator R-GECO1^13^, which was excited at 559 nm spectrally distinct from the absorption spectrum of the Magnet system (Supplementary Fig. S1). Ca^2+^ responses were transiently evoked in the cells immediately after nMagHigh1 and pMagFast1 were activated upon blue light illumination at 473 nm (Fig. 2B). This result demonstrates that blue light-dependent membrane recruitment of pMagFast1-Gα_q_ induces the Ca^2+^ release in the cells. In contrary, cells in the absence of the light stimulation failed to detect any Ca^2+^ response, implying the excitation light (559 nm) for R-GECO1 did not perturb the interaction of the Magnet system as expected from its absorption spectrum (Fig. 2C and Supplementary Fig. S1). There was not any blue light-dependent response observed in the negative control that cells only expressing R-GECO1 (Supplementary Fig. S3A,B). We also did not detect any Ca^2+^ signal under the blue light illumination when the C71S substitutions were employed to this approach, which well explained the light dependence of this approach (Supplementary Fig. S3C,D).

Next we compared the Magnet-based approach with the corresponding Ca^2+^ modulating tool of optoXRs called opto-α_1_AR. Live cell imaging was conducted after R-GECO1 and opto-α_1_AR were coexpressed in HEK 293 cells. Similarly, 473 nm laser light was illuminated to the specially designated regions as the stimulation source of opto-α_1_AR. We recorded the time-lapse cytosolic fluorescence intensity of R-GECO1. As a result, the response of R-GECO1 was observed with or without 473 nm light illumination in opto-α_1_AR (Fig. 2D,E). This experiment yields that opto-α_1_AR is activated by the excitation light of R-GECO1 because rhodopsin covers a broad absorption band, thereby resulting in undesired Ca^2+^ oscillations.

To evaluate the repeatability of this approach, 473 nm laser light was illuminated to the same cell scheduling 5–10 minutes interval for a total of 100 minutes and every time Ca^2+^ signal was captured by R-GECO1 (Fig. 2F,G). The blue light-dependent oscillations show that the regulation of the membrane recruitment of Gα_q_ and the subsequent release of Ca^2+^ can be repeatedly manipulated with blue light without losing the efficiency. We further certify that the manipulation of intracellular Ca^2+^ level is spatially photo-regulated (Supplementary Fig. S4). Upon blue light illumination, the fluorescence intensity of R-GECO1 in the specified cell was increased, indicating a higher intracellular Ca^2+^ level upon blue light stimulation. While the fluorescence brightness of R-GECO1 generally remained unchanged in the other cells without exposure to blue light, showing that the present system is suitable to space-resolved activation of Ca^2+^ release. Additionally, the amplitude of calcium spikes after blue light illumination showed comparable to that elicited by ligand-induced activation of endogenous histamine receptor (Supplementary Fig. S5).

By the direct comparison between the Magnet-based approach and opto-α_1_AR, we conclude that our strategy affords advantage in selectable usage of photoswitchable dimerization systems having narrow absorption spectrums, such as the Magnet system in this case, which indeed leads to the incorporation with R-GECO1 rending it more implementable for Ca^2+^ imaging.

### Red light-induced Ca^2+^ release

Another advantage of the present strategy over the optoXRs is that Gα_q_ can also be manipulated by different wavelength of light via replacing the Magnet system to other types of dimerization system.

**Figure 3:**
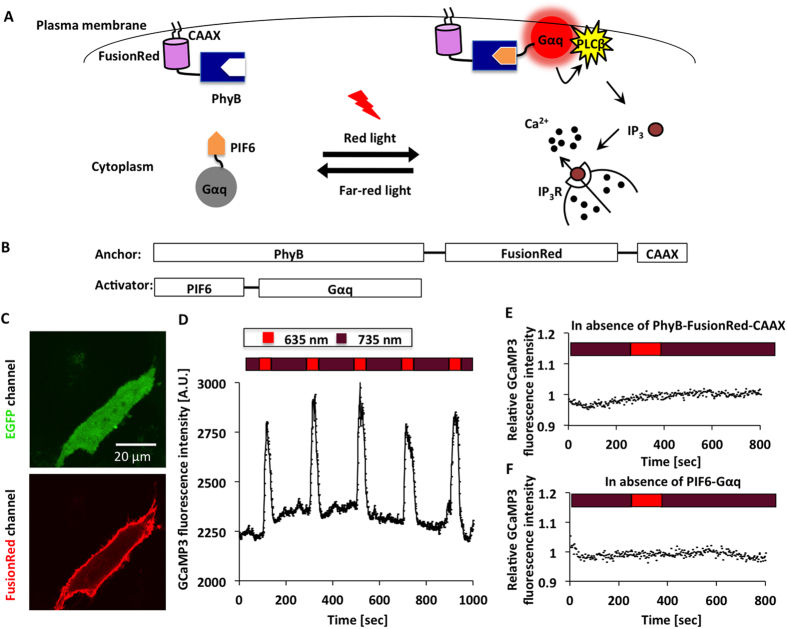
Control of Ca^2+^ release with red light. (**A**) Red light-inducible dimerization of the PhyB/PIF6 system allows the plasma membrane recruitment of Gα_q_ and triggers cytosolic Ca^2+^ release. (**B**) Constructs for PhyB-FusionRed-CAAX and PIF6-Gα_q_. (**C**) Homogeneous cellular diffusion of GCaMP3 in HeLa cell (upper panel) and the plasma membrane localization of PhyB-FusionRed-CAAX (lower panel). (**D**) Ca^2+^ imaging of the PhyB/PIF6-based approach with pulsed red light illumination. Time-lapse fluorescence imaging showed that repeatable response of GCaMP3 was evoked upon activation light at 635 nm (red bars), 735 nm far-red (deep red bars) illumination was used as the inactivation light. (**E**) Negative control in the absence of PhyB-FusionRed-CAAX did not show red light-dependent response. (**F**) Negative control in the absence of PIF6-Gα_q_ did not show red light-dependent response.

Here we present another optogenetic approach to manipulate Gα_q_ based on the red light-inducible PhyB/PIF6 system^14^,^15^. The PhyB/PIF6 system is sensitive to red light for binding, and shows low binding affinity in darkness or under far-red illumination. FusionRed-tagged PhyB is anchored to the plasma membrane by tethering to CAAX while PIF6 linked with Gα_q_ is targeted to the cytoplasm. After red light stimulation, the plasma membrane-anchored PhyB binds to the Gα_q_-linked PIF6, and thus leads to the translocation of PIF6-Gα_q_ from the cytoplasm to the plasma membrane, where the Gα_q_ activates PLCβ and subsequently evokes the Ca^2+^ releasing to the cytosol. When under far-red condition, PIF6-Gα_q_ dissociates from the plasma membrane to the cytoplasm, blocking the Ca^2+^ release (Fig. 3A).

For confocal imaging of the plasma membrane translocation of the PhyB/PIF6-based photoswitch, PhyB-FusionRed-CAAX and PIF6-mYFP-Gα_q_ were transfected to HeLa cells. Upon stimulation with red light (635 nm), the calculated fluorescence intensity of mYFP in the cytosol was decreased, while it was increased in the plasma membrane (Supplementary Fig. S6 and Supplementary movie S1), suggesting the red light-induced recruitment of PIF6-mYFP-Gα_q_ to the plasma membrane. Previous researches show that the dissociation of PhyB/PIF6 interaction not only occurs under far-red illumination but also takes place in darkness with very likely different kinetics^14^,^15^, and it is clear that far-red light-induced PhyB/PIF6 dissociation is much faster than the dark reversion rate (Supplementary Fig. S6C,D).

We employed GCaMP3^16^ as the Ca^2+^ indicator and excited it at 473 nm to avoid the interference to the PhyB/PIF6 interaction. PhyB-FusionRed-CAAX, PIF6-Gα_q_ and GCaMP3 were transfected to HeLa cells. It is evident that GCaMP3 was homogeneously dispersed in the cytoplasm and the PhyB domain was anchored to the plasma membrane (Fig. 3C). During the Ca^2+^ imaging with GCaMP3 at 473 nm, far-red (735 nm) and red (635 nm) light was alternatively used to control the dissociation and association of PhyB/PIF6. In accordance with the result of the Magnet-based approach, Ca^2+^ signal could be repeatedly evoked in the cells upon red light illumination at 635 nm (Fig. 3D). Negative controls in the absence of PhyB-FusionRed-CAAX or PIF6-Gα_q_ did not show any red light-dependent Ca^2+^ response (Fig. 3E,F). These results demonstrate that our strategy allows selective utilization of different photoswitchable dimerization systems to manipulate the membrane recruitment of Gα_q_ and light-induced Ca^2+^ release.

### Red light-induced cAMP increase through membrane recruitment of Gα_s_

**Figure 4:**
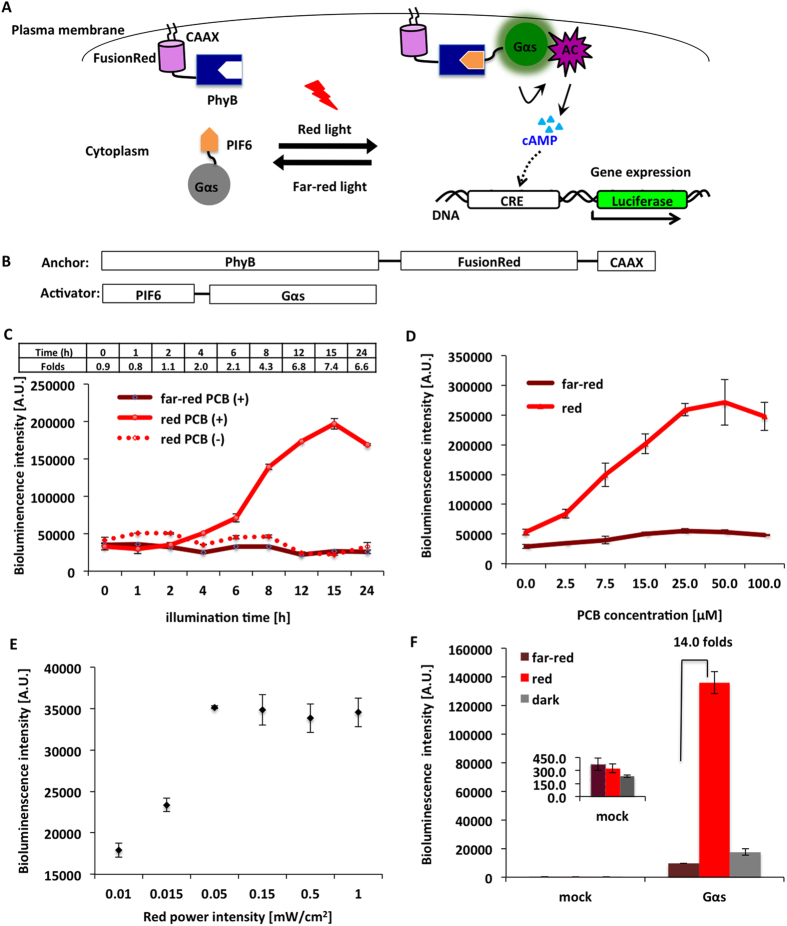
Regulation of cAMP increase with red light. (**A**) Red light-inducible dimerization of the PhyB/PIF6 system allows Gα_s_ to translocate to the plasma membrane and trigger cAMP release owning to the activation of adenylyl cyclase (AC). (**B**) Constructs for PhyB-FusionRed-CAAX and PIF6-Gα_s_. (**C**) Time-lapse luminescence intensity of samples under red illumination at 660 nm (red) or far-red illumination at 735 nm (far-red), with (PCB (+)) or without (PCB (−)) addition of PCB. The increment of luminescence intensity was only observed in the samples under red illumination in the presence of PCB. The upper table means the normalized intensity ratio changes between red PCB (+) and far-red PCB (+). (**D**) Relationship between PCB concentration and the luminescence intensity. (**E**) Power dependency of the luminescence intensity under illumination at 660 nm. (**F**) Result of the bioluminescence assay after optimization of several parameters including PCB concentration (50 μM), illumination time (14 hours) and light power (red: 0.15 mW/cm^2^; far-red: 1.5 mW/cm^2^). The inset is a partially enlarged view of mock, which means a negative control in the absence of PhyB-FusionRed-CAAX. The error bars indicate standard deviation from three individual samples (n = 3).

Varieties of Gα appropriate different downstream effectors in the plasma membrane. As already mentioned, the manipulation of the Gα_q_ leads to the release of cytosolic Ca^2+^. We sought to extend our strategy by the replacement of PIF6-linked Gα_q_ to PIF6-linked Gα_s_ (Fig. 4A). Gα_s_ activates the downstream adenylyl cyclase (AC) after its localization at the plasma membrane and catalyzes the conversion of adenosine triphosphate (ATP) to 3′,5′-cyclic AMP (cAMP) and pyrophosphate^3^,^17^,^18^.

We employed cAMP response element (CRE) driven secretory luciferase construct P_CRE_-luc^19^ as a reporter to test whether light-actuated recruitment of Gα_s_ to the plasma membrane will increase the cAMP level in living cells. cAMP regulates the transcription of the downstream luciferase gene via a conserved gene promoter element CRE (Fig. 4A). Theoretically, a higher intracellular cAMP level leads to a greater expression of luciferase, which can be sensitively measured by the luminescence intensity.

PhyB-FusionRed-CAAX, PIF6-Gα_s_ and P_CRE_-luc were transfected to HEK293 cells, and the cells were kept in a dark incubator for protein expression before exposure to light. We measured the cAMP level by tracing the luminescence intensity of the CRE-driven gene expression products. The luminescence intensity of samples under the far-red light (735 nm) condition remains constant (Fig. 4C), which reveals far-red illumination kept the intracellular cAMP in a roughly invariant level. Conversely, the luminescence intensity of the samples under the red light (660 nm) condition significantly increased after the beginning of the illumination and reached a maximum value in about 15 hours. This result indicates that red light-triggered recruitment of Gα_s_ generates a higher intracellular cAMP level that is sufficient to facilitate the gene expression.

The incorporation of cofactor phytochromobilin (PΦB) or phycocyanobilin (PCB) is required for the light sensitivity of PhyB^20^. As expected, the luminescence intensity of samples in absence of cofactor PCB did not increase even under red light illumination (Fig. 4C). Further analysis about the correlation between PCB concentration and the PhyB/PIF6-induced cAMP level suggested that 50 μM is preferable under the conditions we used (Fig. 4D). Moreover, we measured the red power dependency against the luminescence intensity. The result indicates that the cAMP level is precisely fine-tuned by varying the red light power between 0.01 mW/cm^2^ and 1 mW/cm^2^ (Fig. 4E).

Finally optimization of several parameters including PCB concentration, illumination time and light power allowed a 14-fold bioluminescence intensity difference between the samples under red (660 nm) and far-red (735 nm) light conditions (Fig. 4F). Besides, the samples kept in darkness showed a similar result to those illuminated by far-red light (Fig. 4F), which is consistent with the fact that both dark and far-red conditions make PhyB binding with its partner PIF6 in a low affinity. As a negative control, cells without expressing PhyB-FusionRed-CAAX showed very faint luminescence and appeared no appreciable difference between red and far-red conditions (Fig. 4F). Additionally, we showed that the red light-induced cAMP increase with the PhyB/PIF6-based Gα_s_ system is more significant than cAMP increase stimulated by endogenous GPCR ligands and the effect is approximately equivalent to the result when 5 μM foskolin was tested (Supplementary Fig. S7). Based on all the calculated results, it is proved that optical manipulation of Gα using our strategy is not merely applicable to Gα_q_ but also suited for Gα_s_.

## Discussion

In this study, we developed a strategy for light-actuated recruitment of Gα_q_ and Gα_s_ to the plasma membrane using either the Magnet system or the PhyB/PIF6 system. We demonstrated the achievement of light-dependent regulation of second messengers including Ca^2+^ and cAMP in mammalian cells.

Our strategy provides multiple selections of dimerization system to manipulate Gα in order to meet the demands of different applications. We demonstrate that the wavelength used in light-triggered recruitment of the Gα_q_ can be easily shifted from blue (473 nm) to red (635 nm) by replacing the dimerization systems. Both two approaches achieve temporal and repeatable regulation of cytosolic Ca^2+^ release upon photoirradiation. These two different dimerization systems have their own features. The Magnet system has high interaction affinity, fast switch-off kinetic, and an exogenous addition of cofactor is not required. The PhyB/PIF6 system should be superior when applied to *in vivo* studies because of the better tissue penetration of red light. Regardless of which approach is used, it should be emphasized that the most remarkable characteristic for manipulation of Gα_q_ with the Magnet system and the PhyB/PIF6 system is the competitive advantage over the opto-α_1_AR. Opto-α_1_AR getting broad absorption band suffers from the perturbation by the excitation light for Ca^2+^ indicators. The use of the Magnet system or the PhyB/PIF6 system that has narrow absorption band overcomes this fundamental difficulty. These photoswitching systems are supposed to free up vacant spectrum that provide more selection of fluorescent proteins for labeling. This distinct spectral property also can be harnessed to combine with other optogenetic tools such as channelrhodpsin-2 (refs 21,22).

The successful manipulation of both Gα_q_ and Gα_s_ demonstrates that our strategy is feasible to optically manipulate different classes of Gα. Light-dependent control of Ca^2+^ and cAMP has been achieved through the corresponding activation of Gα_q_ and Gα_s_. Optical regulation of these second messenger molecules provides extensive applications to mediate biological process. Furthermore, optical recruitment of other classes of Gα as well as Gα_q_ and Gα_s_ to the plasma membrane may also be applicable by indiscriminately apply this strategy, and the property of ‘auto-activation’ upon the plasma membrane recruitment can be shared to other classes of Gα, because they are generally believed to carry out signal functions at the plasma membrane. Therefore, diversified downstream pathways presumably can be regulated so as to mediate various patterns of signaling events in mammalian cells.

In summary, we provide a strategy to construct highly versatile approaches to optically manipulate the Gα. Various types of Gα can be recruited to the plasma membrane using different dimerization systems so as to reach the activation. As the functional application of this strategy, it enables optical switchable regulation of different second messengers in mammalian cells.

## Methods

### DNA constructions

The humanized genes encoded PhyB construct harboring tandem PAS (1–908) and 100-residue N-terminal phytochrome binding domain of PIF6 were synthesized by Eurofins Genemics (Tokyo, Japan). cDNAs encoding human Gα_q_ and Gα_s_ were gifted from Dr. Putyrski. We used the C9S and C10S mutants in Gα_q_. Because the wild type Gα_q_ is palmitoylated at these two cysteine residues for membrane localization^23^,^24^, and these two mutations prevent membrane association of the protein^25^. The Q209L mutant lacking the constitutive GTPase activity in Gα_q_^26^ was also used to improve the present Gα_q_-based optogenetic approach. The present mutagenesis was performed with an overlap extension technique and Multi Site-Directed Mutagenesis Kit (MBL, Nagoya, Aichi, Japan) according to the manufacturer’s instructions.

All other plasmid constructions appeared in this study were created by standard molecular biology techniques and separately subcloned into the mammalian expression vector pcDNA3.1. All cloning enzymes were obtained from Takara Biomedical (Tokyo, Japan) and used according to the manufacturer’s instructions. The constructions were confirmed by sequencing the cloned fragments.

### Protein purification and spectral analysis

nMagHigh1 with an N-terminal six-residue histidine tag was expressed in *Escherichia coli* DH5α cells with the pCold I vector and cultured in 200 mL LB medium containing 40 mg/ml of ampicillin. The bacterial cells were grown at 37 °C until they reached a density of approximately OD_600_ = 0.5. Protein expression was induced by addition of isopropyl *β*-d-1-thiogalactopyranoside (IPTG) at a final concentration of 0.1 mM following a temperature downshift from 37 °C to 15 °C. The bacterial cells were cultured for 48 hours. The histidine-tagged proteins were purified using TALON Spin Columns (Clontech, Palo Alto, CA) and eluted with an imidazole solution (500 mM imidazole, 50 mM sodium phosphate, 300 mM NaCl, pH 7.0). Absorption spectrometry was performed at room temperature using an Evolution Array spectrophotometer (Thermo Scientific, Waltham, MA, USA).

### Cell culture

COS-7 and HEK293 cells were cultured at 37 °C under 5% CO_2_ in Dulbecco’s Modified Eagle Medium (DMEM; Invitrogen). HeLa cells were cultured under 5% CO_2_ in Μinimum Essential Medium Eagle (MEM; SIGMA). Both mediums were supplemented with 10% fetal bovine serum (GIBCO, Carlsbad, CA, USA), 100 unit/ml of penicillin and 100 μg/ml of streptomycin (GIBCO). These cells were used for translocation assay, Ca^2+^ imaging and bioluminescence assay.

### TIRF imaging

To conduct the plasma membrane-translocation assay of the activator construct using TIRF imaging, COS-7 cells were plated at 1.0 × 10^4^ cells per dish on glass-bottomed dishes and cultured for 24 hours at 37 °C in 5% CO_2_. The cells were transfected with cDNAs encoding DsRedEx2-pMagFast1-Gα_q_ and nMagHigh1-mKikGR-CAAX at a 1:1 ratio using X-tremeGENE 9 DNA transfection reagent (Roche Diagnostics GmbH, Mannheim, Germany) according to manufacturer’s protocol. The total amount of DNA was 1 μg per dish. Twenty-four hours after transfection, the medium was replaced by the DMEM culture medium supplemented with 10% FBS. The cells were maintained for 24 hours at 28 °C. Before imaging, the culture medium was replaced with Hanks’ Balanced Salt Solution (HBSS, Grand Island Biological Co., Grand Island, NY, USA) containing 10 mM HEPES. Imaging was performed at room temperature with a 100× oil objective on the stage of an ECLIPSE Ti TIRF microscope (Nikon, Tokyo, Japan). Fluorescence images of DsRedEx2 were taken using an optically pumped semiconductor laser at 561 nm (Coherent, CA, USA). Blue light illumination was conducted using an optically pumped semiconductor laser (488 nm) at 1 mW for 50 ms.

### Live cell imaging

The 35 mm glass-bottomed dishes (AGC TECNO GLASS Co., Shizuoka, Japan) were coated with poly-l-lysine (Sigma-Aldrich Co., Missouri, USA) at room temperature for 60 minutes, washed twice with 2 ml Milli-Q for use. In Fig. 2 and Supplementary Fig. S3, HEK293 cells were plated at approximately 2.0 × 10^4^ cells/dish and cultured for 24 hours at 37 °C in 5% CO_2_. Cells were transfected with cDNAs encoding nMagHigh1-mKikGR-CAAX, pMagFast1-Gα_q_ and R-GECO1 or substitutions using X-tremeGENE 9 DNA transfection reagent. Twenty-four hours after transfection, the culture media were replaced with HBSS containing 10 mM HEPES. Live cell imaging was conducted at 37 °C with a heated stage adaptor (Tokai Hit Co., Fujinomiya, Shizuoka, Japan). Fluorescence images of mKikGR and R-GECO1 were taken using a solid-state laser at 515 nm (Coherent) and a laser diode at 559 nm (NTT electronics, Yokohama, Japan) respectively. Blue light illumination was completed with a Laser diode at 473 nm.

In Fig. 3, HeLa cells were plated in 35 mm glass bottom dish (MATSUNAMI, Tokyo, Japan) and seeded for 24 hours, achieving to approximately 70% confluence of the plate. cDNAs encoding PhyB-FusionRed-CAAX, PIF6-Gα_q_ and GCaMP3 were transfected to cells at a ratio 3:1:1 using the Lipofectamine 3000 Transfection Kit (Invitrogen, CA, USA) for 24 hours at 37 °C. The total amount of DNA was 0.5 μg. The cells were further incubated for 12 hours at 28 °C. PCB (Frontier Scientific, Logan, UT, USA) was added to the cells under dark condition to give a final concentration of 20 μM and incubated another 60 minutes. Then the culture media were washed and replaced with HBSS. Live cell imaging was conducted at 28 °C with the heated stage adaptor. Fluorescence images of FusionRed and GCaMP3 were taken using a solid-state laser at 559 nm (NTT electronics, Yokohama, Japan) and a laser diode at 473 nm (Olympus, Tokyo, Japan) respectively. Red light illumination was completed with a laser diode at 635 nm (Olympus). LED array (735 nm ± 20 nm, 1.5 mW/cm^2^) was used as the inactivation light. In Supplementary Fig. S6, to monitor the plasma membrane recruitment of PIF6-Gα_q_, mYFP (Q70K) was inserted between PIF6 and Gα_q_. Translocation assay was performed under a same condition after PhyB-FusionRed-CAAX and PIF6-mYFP-Gα_q_ were transfected to HeLa cells at a 9:1 ratio.

FV1200 confocal laser scanning microscope (Olympus) and 60× oil immersion objective were used in live cell fluorescence imaging and translocation assay.

### Bioluminescence assay

HEK 293 cells were plated at approximately 1.0 × 10^5^ cells/well in a 24-well plate (AGC TECNO GLASS Co., Shizuoka, Japan), and cultured for 24 hours at 37 °C in 5% CO_2_. cDNAs encoding PhyB-FusionRed-CAAX, PIF6-Gα_s_ and the reporter pGL4.29 [luc2P/CRE/Hygro] were transfected to cells at a ratio 3.5:0.5:1 using the Lipofectamine 3000. The total amount of DNA was 0.5 μg/well. Then samples were covered with foil to ensure dark conditions and kept in 37 °C incubator for 12 hours, an additional 12 hours incubation at 28 °C was executed for enough protein accumulation and better membrane localization. PCB was dissolved in DMEM and added to the samples half an hour before illumination. We used LED light sources (CCS Inc., Kyoto, Japan) for red (660 ± 20 nm) and far-red (735 ± 20 nm) light illumination respectively. The culture medium was removed after a period of illumination. The cells were treated with 200 μl/well passive lysis buffer (Promega, Madison, WI, USA) for 15 minutes at room temperature. 500 μl HBSS culture medium containing 200 μM d-luciferin pottasium salt (Wako Pure Chemistry Industries, Ltd., Osaka, Japan) was prepared as a substrate. Bioluminescence measurements were immediately performed after mixing the d-luciferin solution with the lysed cells using a Glomax 20/20 Luminometer at room temperature (Promega).

## Additional Information

**How to cite this article**: Yu, G. *et al.* Optical manipulation of the alpha subunits of heterotrimeric G proteins using photoswitchable dimerization systems. *Sci. Rep.* **6**, 35777; doi: 10.1038/srep35777 (2016).

## Supplementary Material

Supplementary Information

Supplementary Movie S1
