# Supplementary material for: Optical manipulation of the alpha subunits of heterotrimeric G proteins using photoswitchable dimerization systems
Source: Sci Rep. 2016 Oct 21;6:35777. doi: 10.1038/srep35777 (PMC5073340; doi:10.1038/srep35777)
Supplement: Supplementary Information [file srep35777-s1.pdf]

Optical manipulation of the alpha subunits of heterotrimeric G proteins using photoswitchable dimerization systems

Gaigai Yu[+], Hiroyuki Onodera[+], Yuki Aono, Fuun Kawano, Yoshibumi Ueda, Akihiro Furuya, Hideyuki Suzuki and Moritoshi Sato[*]

Graduate School of Arts and Sciences, The University of Tokyo, Komaba, Meguro-ku, Tokyo 153-8902, Japan.

[+] These authors contributed equally to this work.

[*] To whom correspondence should be addressed.

Tel: +81-3-5454-6579; Fax: +81-3-5454-6579;

E-mail: cmsato@mail.ecc.u-tokyo.ac.jp

Supplementary Information

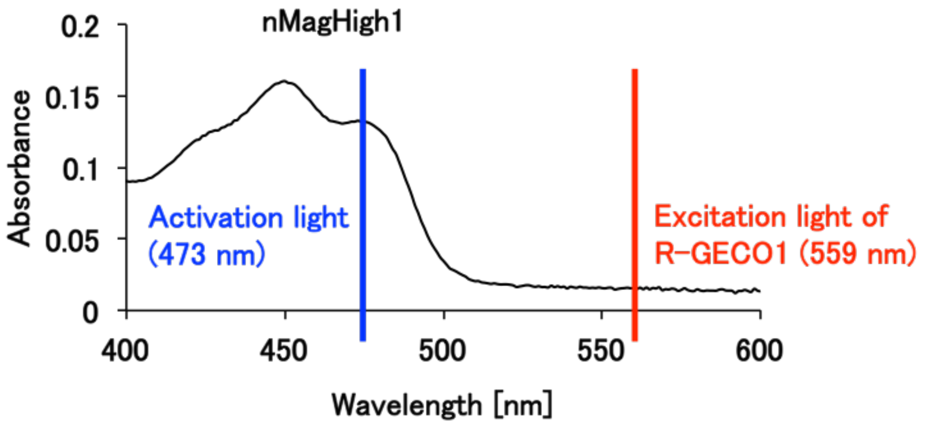

Supplementary Figure S1. Spectral property of nMagHigh1. The absorption spectrum of purified nMagHigh1 protein. Blue line stands for the activation light we used for dimerization of the Magnet system. Red line stands for the excitation light we used for imaging R-GECO1 fluorescence.

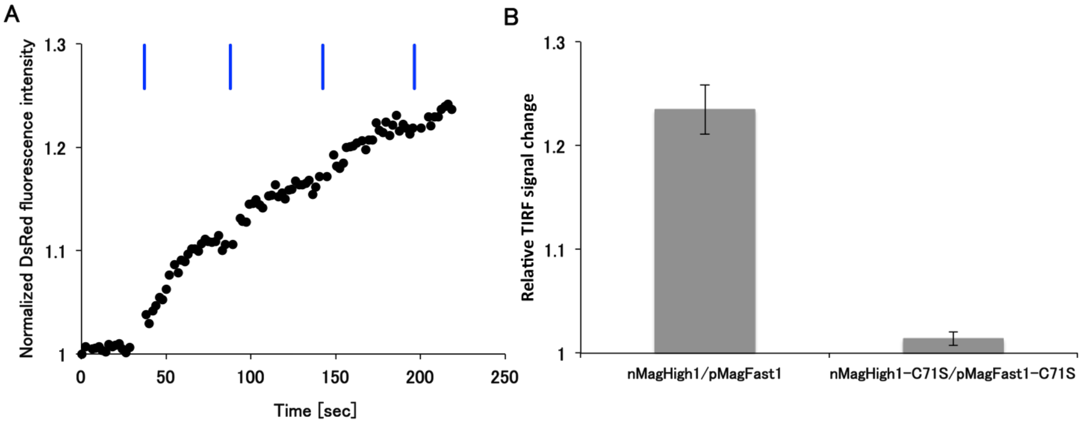

Supplementary Figure S2. TIRF translocation assay. (A) nMagHigh1-mKikGR-CAAX and DsRedEx2-pMagFast1-Gα_q were coexpressed in COS-7 cells. The fluorescence intensity was significantly increased after blue light stimulation at 488 nm (blue bar). (B) The Magnet system-based probe (nMagHigh1/pMagFast1) showed a remarkable TIRF signal change upon the blue light illumination while negative mutants with C71S substitutions (nMagHigh1-C71S/pMagFast1-C71S) showed no significant TIRF signal change after the blue light stimulation.

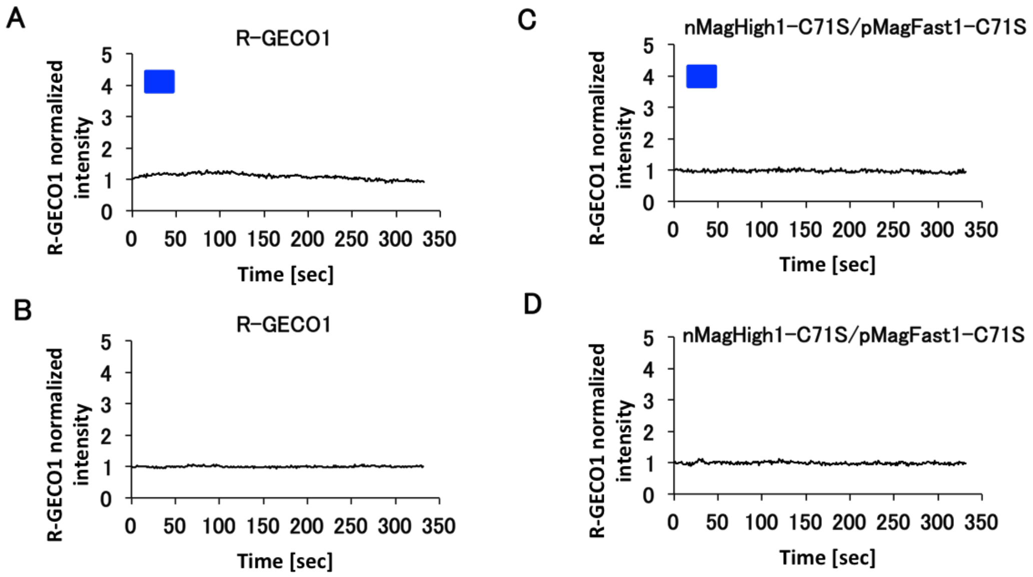

Supplementary Figure S3. Negative controls of blue light-induced Gα_q system. When only the Ca^{2+} indicator R-GECO1 was transfected to cells, no signal was evoked with (A) or without (B) blue light stimulation. By introducing the C71S mutations that impair the photoswitching dimerization of the Magnet system, Ca^{2+} signal was not evoked with (C) or without (D) blue light stimulation. Blue bars indicate blue light illumination at 473 nm.

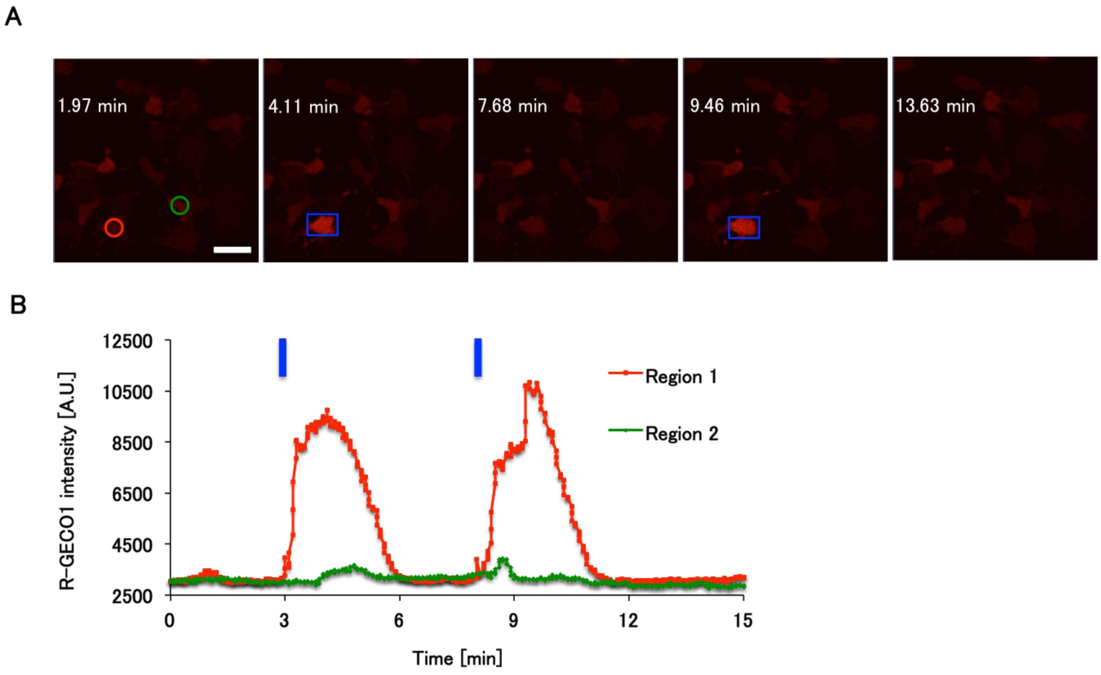

Supplementary Figure S4. Spatial control of blue light-induced Ca^{2+} release. (A) Fluorescence imaging of spatial control of blue light-induced Ca^{2+} release. HEK293 cells were expressed with nMagHigh1-mKikGR-CAAX, pMagFast1-Gα_q and R-GECO1. Only the specified region (blue rectangle) illuminated with blue light at 488 nm showed Ca^{2+} release. Scale bar 50 μm. (B) The time-lapse fluorescence intensity of R-GECO1 in region 1 and region 2, which are marked respectively as red and green circles in (A) at 1.97 min.

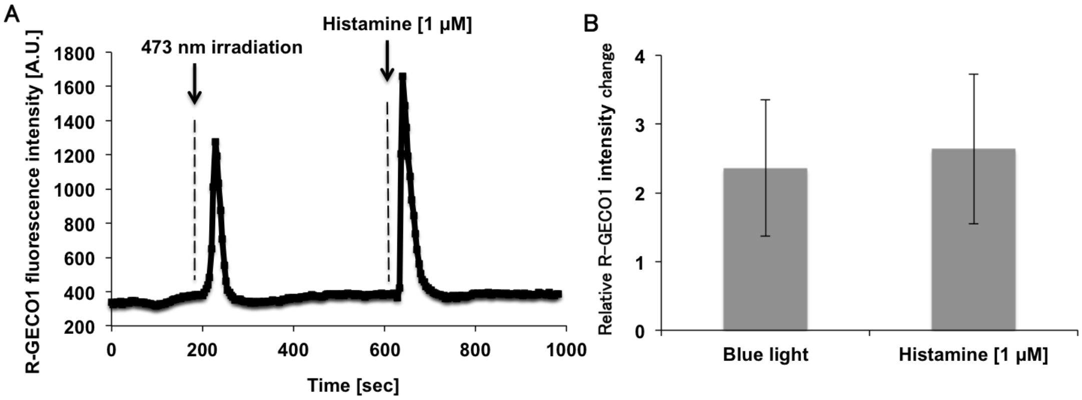

Supplementary Figure S5. Comparison of blue light-induced Ca^{2+} response with histamine-induced Ca^{2+} response. (A) Measurement of fluorescence intensity of R-GECO1 upon different stimulations with blue light (473 nm, 5 mW/cm^2, 10 sec) and histamine (1 μM). (B) Averaged amplitude of R-GECO1 fluorescence intensity by blue light illumination (n=10) or histamine stimulation (n=12). Error bar represents standard deviation. The amplitude of calcium spikes after blue light illumination showed comparable to that elicited by histamine. Student's test ($P > 0.05$).

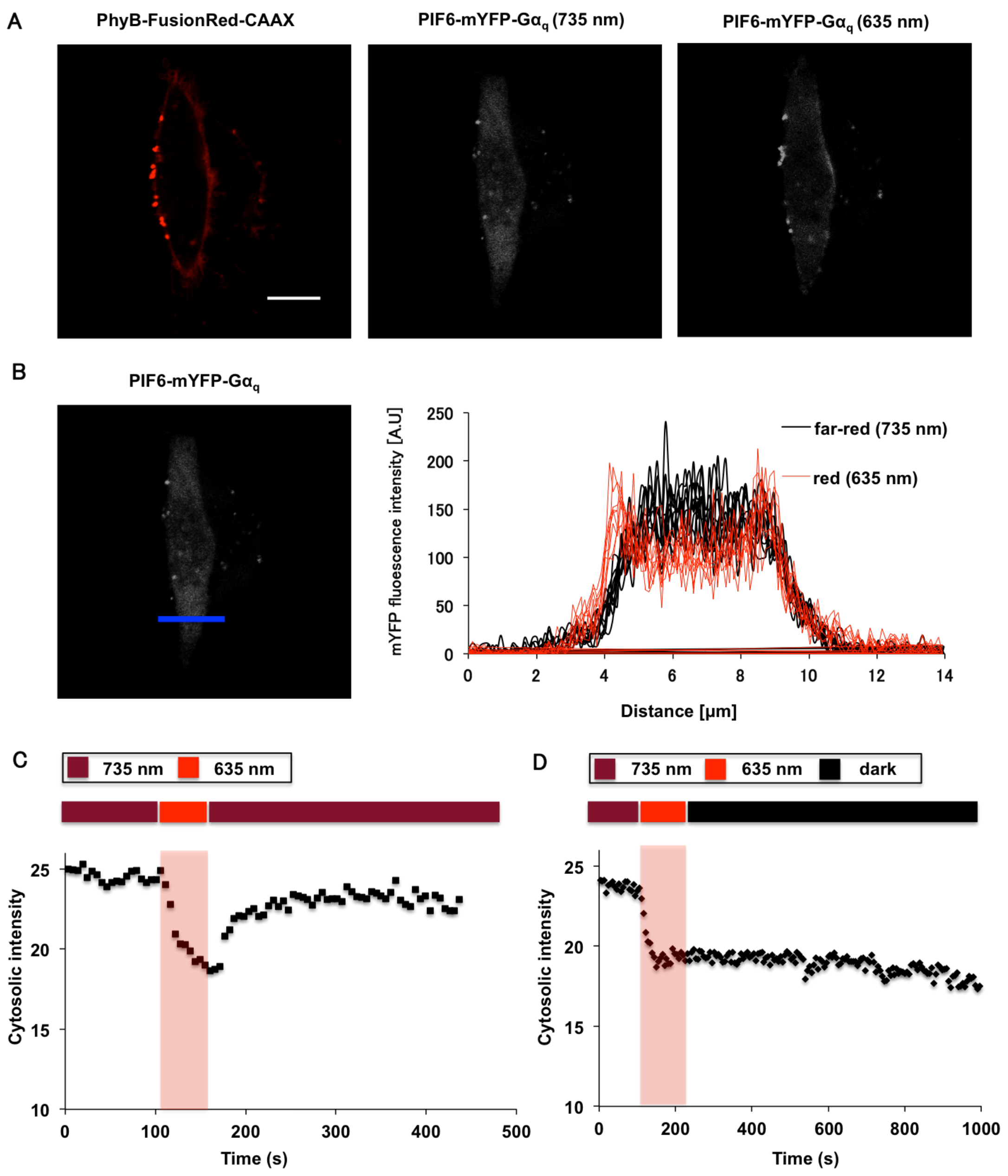

Supplementary Figure S6. Translocation of the PhyB/PIF6-based photoswitching system. (A) Expression of PhyB-FusionRed-CAAX and PIF6-mYFP-Gα_q. Scale bar, 10 μM. The fluorescence intensity of mYFP increased in the plasma membrane upon stimulation with red light at 635 nm, demonstrating the translocation of PIF6-mYFP-Gα_q to the plasma membrane. (B) Line profiles (right panel) represent fluorescence intensity of mYFP measured along the blue line of the PIF6-mYFP-Gα_q image (left panel). (C) Cytosolic fluorescence of mYFP was immediately increased after far-red illumination suggesting that far-red induced PhyB/PIF6 dissociation quickly. (D) There was no cytosolic fluorescence increase in the short period time under dark condition.

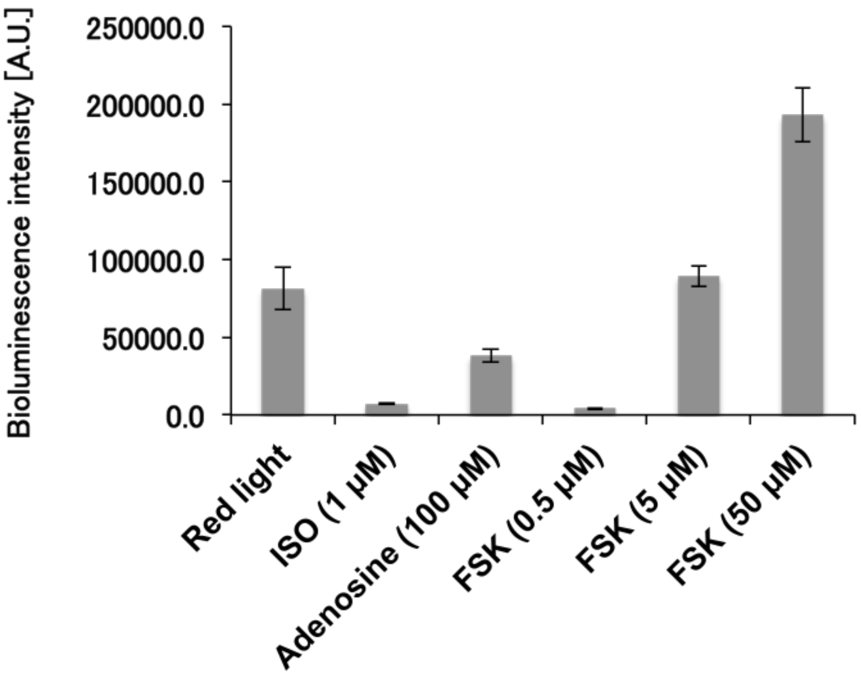

Supplementary Figure S7. Comparison of red light-induced cAMP level with native GPCR ligands and forskolin. HEK 293 cells transfected with PhyB-FusionRed-CAAX, PIF6-Gα_s and reporter gene (P_{CRE}-luc), and the bioluminescence intensity was measured 12 hours after red light illumination (660 nm, 0.15 mW/cm^2). While other cells were transfected with reporter only but cheated with 1 μM isoprenaline (ISO), 100 μM adenosine and different concentration (0.5, 5, 50 μM) of forskolin (FSK). The error bars indicate standard deviation from three individual samples.

Supplementary Movie S1. Translocation of the PhyB/PIF6-based photoswitching system. PhyB-FusionRed-CAAX and PIF6-mYFP-Gα_q were transfected to HeLa cells. Confocal microscopy of the HeLa cell showes that mYFP is dispersed in the cytoplasm under far-red (735 nm) illumination. Upon turning on the red light (635 nm), the fluorescence intensity of mYFP in the cytosol was rapidly decreased and the intensity in the plasma membrane was increased, which reveals the plasma membrane recruitment of PIF6-mYFP-Gα_q. Turning off the red light and exposure to far-red illumination allowed the fast release of PIF6-mYFP-Gα_q to the cytosol.
